# Postoperative Outcomes with and without Neoadjuvant Denosumab in Grade-III Giant Cell Tumor of the Proximal Femur: A Comparative Retrospective Study

**DOI:** 10.1055/s-0045-1809397

**Published:** 2025-07-10

**Authors:** Sheikh Muhammad Ebad Ali, Badaruddin Sahito, Hina Khan, Awais Abro, Sunel Kumar, Muhammad Usman Ali

**Affiliations:** 1 Department of Trauma & Orthopedics, Chelsea & Westminster Hospital, London, United Kingdom; 2 Department of Trauma & Orthopedics, Dow University of Health Sciences (DUHS), Sindh, Pakistan; 3 Karachi Medical and Dental College (KMDC), Sindh, Pakistan; 4 Ruth KM Pfau Civil Hospital Karachi, Sindh, Pakistan

**Keywords:** bone neoplasms, denosumab, giant cell tumor, hip, proximal femoral fractures, denosumab, fraturas proximais do fêmur, neoplasias ósseas, quadril, tumores de células gigantes

## Abstract

**Objective:**

To retrospectively compare the impact of using neoadjuvant denosumab for Campanacci grade-III giant cell tumor (GCT) of the proximal femur involving the hip joint.

**Methods:**

We retrospectively reviewed 18 cases of Campanacci grade-III GCT of the proximal femur receiving surgery between January 2014 and December 2019 from our hospital. One group of 10 patients received weekly neoadjuvant denosumab 120 mg for 4 weeks, while the other group of 8 patients did not receive denosumab before surgery. Two patients were subjected to intralesional curettage while the others received resection and hip arthroplasty. Comparisons were made using unpaired *t* -test and Fisher's exact test. Functional outcomes were assessed by revised Musculoskeletal Tumor Society (MSTS) score and Harris Hip Score (HHS) at 6 weeks, 6 months, and 12 months of follow-up, as well as incidence of recurrence.

**Results:**

The comparison of the mean MSTS scores of the denosumab and non-denosumab groups was as follows: 24.0 ± 6.5 versus 20.0 ± 6.0 ( *p* = 0.04) at 6 weeks respectively; 26.0 ± 5.0 versus 23.0 ± 0.67 ( *p* = 0.04) at 6 months respectively; and 28.8 ± 1.7 versus 29.5 ± 0.33 ( *p* = 0.35) at 12 months respectively. The comparison of HHSs between the denosumab and non-denosumab groups was as follows: 61.02 ± 7.36 versus 48.52 ± 3.97 ( *p* = 0.03) at 6 weeks respectively; 81.1 ± 2.97 versus 79.15 ± 3.24 ( *p* = 0.82) at 6 months respectively; and 89.84 ± 3.75 versus 90.05 ± 3.00 ( *p* = 0.38) at 12 months respectively. There was no recurrence.

**Conclusion:**

Denosumab was clinically effective in improving the short-term functional outcomes, but long-term functional outcomes remained similar between the groups. We did not find an increased recurrence rate in the denosumab group.

## Resumo

**Objetivo:**

Comparar retrospectivamente o impacto do uso de denosumabe neoadjuvante em tumores de células gigantes (TCG) de grau III de Campanacci no fêmur proximal com acometimento da articulação do quadril.

**Métodos:**

Avaliamos retrospectivamente 18 casos de TCG de grau III de Campanacci do fêmur proximal que foram submetidos à cirurgia entre janeiro de 2014 e dezembro de 2019 em nosso hospital. Um grupo de 10 pacientes receberam denosumabe neoadjuvante em dose de 120 mg uma vez por semana por 4 semanas, enquanto outro grupo, com 8 pacientes, não recebeu denosumabe antes da cirurgia. Dois pacientes foram submetidos à curetagem intralesional e os demais, à ressecção e artroplastia de quadril. As comparações foram feitas usando o teste *t* não pareado e o teste exato de Fisher. Os resultados funcionais foram avaliados pelo escore revisto da *Musculoskeletal Tumor Society* (MSTS) e pelo escore de quadril de Harris (Harris Hip Score, HSS) em 6 semanas, 6 meses e 12 meses de acompanhamento. A incidência de recidiva também foi determinada.

**Resultados:**

A comparação das médias dos escores MSTS entre os grupos com e sem denosumabe foi de 24,0 ± 6,5 *versus* 20,0 ± 6,0 ( *p* = 0,04) em 6 semanas, respectivamente; 26,0 ± 5,0 *versus* 23,0 ± 0,67 ( *p* = 0,04) em 6 meses, respectivamente; e 28,8 ± 1,7 *versus* 29,5 ± 0,33 ( *p* = 0,35) em 12 meses, respectivamente. A comparação das médias das pontuações no HSS entre os grupos com e sem denosumabe foi de 61,02 ± 7,36 *versus* 48,52 ± 3,97 ( *p* = 0,03) em 6 semanas, respectivamente; 81,1 ± 2,97 *versus* 79,15 ± 3,24 ( *p* = 0,82) em 6 meses, respectivamente; e 89,84 ± 3,75 *versus* 90,05 ± 3,00 ( *p* = 0,38) em 12 meses, respectivamente. Não houve recidiva.

**Conclusão:**

O denosumabe foi clinicamente eficaz na melhora dos desfechos funcionais em curto prazo, mas os desfechos funcionais em longo prazo foram semelhantes entre ambos os grupos. Não observamos aumento na taxa de recidiva no grupo com denosumabe.

## Introduction

Giant cell tumor (GCT) of the bone is a benign but locally aggressive tumor of the end of a long bone. It is characterized by mononuclear stromal cell growth and the presence of many multinucleated giant cells with a homogenous distribution. 1 The distal femur, proximal tibia, and distal radius are the most frequent sites for GCTs in the metaphyseal-epiphyseal region. 1 Giant cell tumor of the proximal femur is a rare entity with higher morbidity due to changes in gait, lifestyle disturbances, risk of pathological fractures, and limitations in daily activities. Moreover, the disease is associated with the younger population, which may be at increased risk of longer poor quality of life. Therefore, GCT of the proximal femur warrants special attention. It has a slight female prevalence and occurs more frequently in the third and fourth decades of life. 2 Grade III is ascribed to GCT with poorly defined margins, cortical bone loss, and invasion of the surrounding soft tissues according to the Campanacci grading system. 3

Denosumab is the only US Food and Drug Administration (FDA)-approved drug for the treatment of GCT. 4 Denosumab decreases the number of multinucleated giant cells in the bone matrix and accelerates the development of the mature rim around the tumor by blocking the receptor activator of nuclear factor-κB (RANK)/RANK-ligand (RANKL) pathway. Denosumab has been employed as a neo-adjuvant therapy method in prior trials. However, the studies showed a substantially larger number of denosumab cycles preoperatively. 5

The present study is the first that aims to investigate the functional and oncological outcomes of neoadjuvant denosumab for Campanacci grade III GCT of proximal femur with surgery. Previously conducted trials on GCT of proximal femur mainly focused upon resection and replacement of proximal femur with endoprosthesis, Wagner prosthesis, total hip arthroplasty, and hemiarthroplasty of the hip. 6 7 These studies did not consider intralesional curettage as an option, except for Wijsbek et al. who performed intralesional curettage for 10 patients but did not use neoadjuvant denosumab. 8 Considering the average age of the population, a joint salvage approach would be more appropriate than prosthetic replacements as these have limited duration, after which loosening of prosthesis may occur. 9 All of the studies performed to date assessed the oncological outcomes by Musculoskeletal Tumor Society (MSTS) score without utilizing other hip scores to evaluate the functional outcome and quality of life after surgery.

## Materials and Methods

### Study Design

The data used in the present study was acquired retrospectively from the record files of the patients who were treated from January 2014 to December 2019 at a tertiary hospital. The patients' data included the name, age, diagnosis with radiographs and biopsy reports, bone involved, management plan, status on last follow-up and contact for correspondence. The patients were individually contacted by the second author for signed informed consent before registration, following the Declaration of Helsinki. The present work received our approval Institutional Review Board under number CHK/DMC/Ortho/Dated 11–22/001.

### Inclusion and Exclusion Criteria

The patients having a diagnosis of Campanacci grade III GCT of proximal femur confirmed on imaging and had received surgery were included in the study. The GCT of the proximal femur included GCT of anatomical landmarks of the head, neck, trochanteric, and subtrochanteric areas. The study population included was stratified into two cohorts according to the use of preoperative subcutaneous denosumab 120 mg injections given once weekly for 4 weeks before proximal femur surgery. All patients who were treated by surgery but did not receive denosumab were stratified into the non-denosumab group, while those who underwent surgery and received denosumab were included in the denosumab group.

Exclusion was done based on skeletal immaturity and missing age from records. Patients who had primary and secondary malignant GCT, grade-I or - GCT, follow-up for less than 12 months, non-compliance with postoperative rehabilitation, less than 4 doses of denosumab, and previous curettage for GCT as well as those who did not provide consent were also excluded from the study.

### Surgical Technique

Selection of any one of the following two methods were made after discussion in the preoperative meeting of the multidisciplinary team of the tumor board, where GCT tumors were reassessed after 4 doses of denosumab.

- Intralesional curettage with hemicortical resection
- Resection and hip arthroplasty.

#### Intralesional Curettage with Hemicortical Resection

**Fig. 1:**
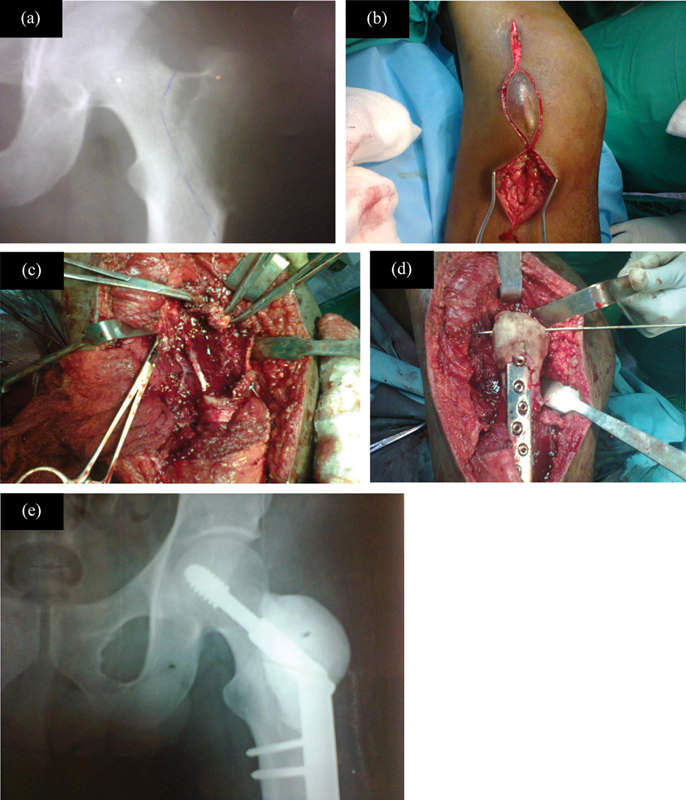
Surgical methods ( **A** ) preoperative X-ray after denosumab therapy ( **B** ) boat-shaped incision ( **C** ) after creating cortical window and high-speed burring curettage ( **D** ) filling with bone allograft, cement with DHS ( **E** ) postoperative X-ray showing DHS and allograft with bone cement.

After consent and counseling and under aseptic measures, through a lateral approach, skin, subcutaneous tissue, and fascia were incised, and the tumor was located. Under the C arm, a cortical window was made by resection of the margins, including the lateral wall of the neck and the greater trochanter. The residual tumor at the medial wall was curetted, and burring was used to clear the remaining tumor and to level the surface. Once the surface became smooth and tumor free, cauterization and hydrogen peroxide were used to burn the micro tumor particles. A dynamic hip screw was placed, and the defect was reconstructed with bone cement. Holes were created in the bone cement and the abductors were reattached. The surgical techniques are shown in Fig. 1 .

After final washing, the wound was closed in layers. Early hip movement was encouraged postoperatively, and weight bearing was resumed once the patients were able to tolerate it. Stitches were removed after 2 weeks, and patients were followed-up fortnightly for 3 months, then monthly for 6 months, thereafter, every 3 months for 2 years and then, biannually afterwards. On each visit, hip movements were assessed and documented on follow-up charts.

#### Resection and Hip Arthroplasty

Under general anesthesia with patient's consent and antiseptic measures, a longitudinal lateral incision in lateral position was made on the skin after evaluation of the radiographs. After incising through the subcutaneous tissue and fascia, the muscles were reflected. Afterwards, the measurements were obtained, and the femur was cut 2 to 3 cm from the margin of the tumor. Wide margin resection of the GCT was performed, and the specimen was obtained for histopathology of specimen margins. After resection, the site was assessed for suitability of Wagner prosthesis.

- Bipolar hemiarthroplasty was performed in patients with pathological femoral neck fracture or damaged calcar femorale.
- We used the Wagner prosthesis with bipolar head in tumors extended into intertrochanteric or subtrochanteric area in cases in which acetabulum cartilage was not involved.
- Total hip arthroplasty with Wagner prosthesis was used in patients with damaged cartilage and calcar femorale.

**Fig. 2:**
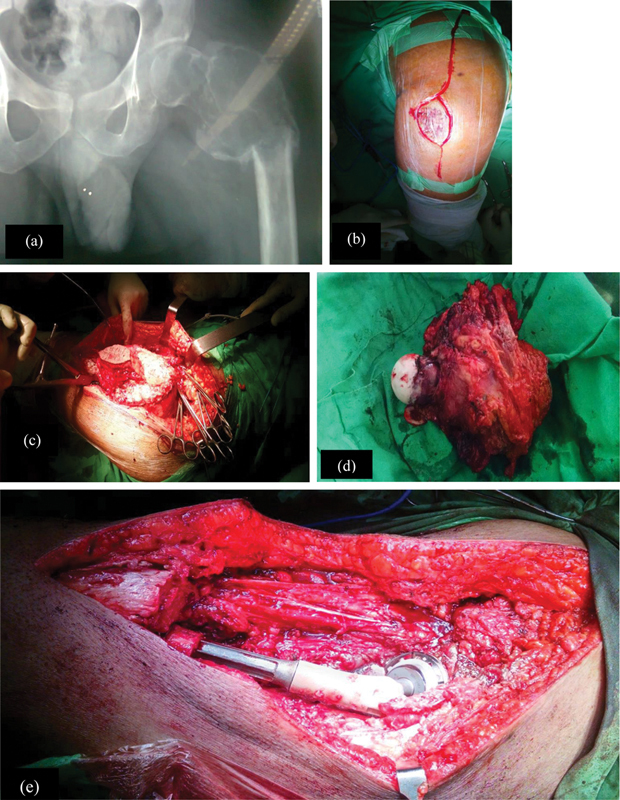
Surgical methods showing ( **A** ) preoperative X-ray after denosumab therapy ( **B** ) boat shaped incision ( **C** ) incision of skin, subcutaneous, and fascia to expose the tumor ( **D** ) Resected specimen of proximal femur ( **E** ) insertion of Wagner prothesis and total hip arthroplasty.

The surgical site was then washed, and the wound was closed in layers. Sterile dressings were applied on alternate days, and sutures were cut after two weeks. Patients were followed-up fortnightly for 3 months, then monthly for 6 months, thereafter, every 3 months for 2 years, and then, biannually afterwards. On each visit, hip movements were assessed and documented on follow-up charts. The surgical techniques are shown in Fig. 2 .

### Comparative Outcomes Analysis

Our primary end point was postoperative outcomes of hip joint, which were assessed at 6 weeks, 6 months and 12 months of follow-ups by the Harris Hip Score (HHS) and the revised MSTS. According to the HHS, 0 to 25% means a poor result, 26 to 50% means a fair result, 51 to 75% means a good result, and 76 to 100% means an excellent result. According to the revised MSTS score for the lower extremities, 0 to 7 means a poor result, 8 to 14 means a fair result, 15 to 22 means a good result, and above 22 means an excellent result, after 6 weeks, 6 months, and 12 months of follow-up; the secondary outcome was incidence of recurrence.

### Statistical Analysis

All descriptive statistics are represented as means with standard deviations for continuous variables. Categorical variables are included as frequencies with percentages. Comparison of baseline characteristics between the two groups was made by either the unpaired *t* -test for the continuous variables or the Fisher's exact test for proportions of two categorical variables as it is more suitable for lower sample-sized studies with a 95% CI for both. The data were analyzed using the IBM SPSS Statistics for Windows software (IBM Corporation), version 22.0. The HHS and the MSTS score are continuous variables, while the incidence of recurrence and complications are categorical variables.

## Results

**Fig. 3:**
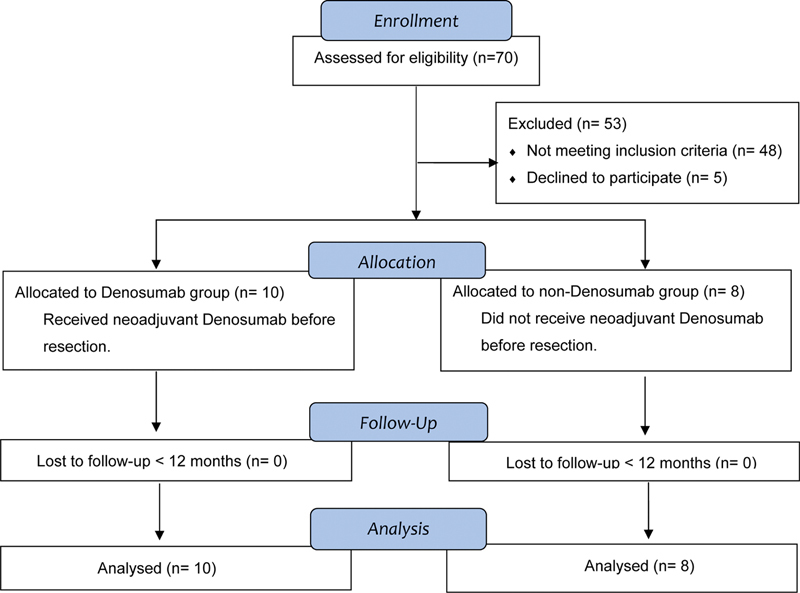
Flowchart for inclusion/exclusion of patients.

None of the study characteristics differ significantly between the denosumab and non-denosumab groups, including mean age (28.6 ± 5.85 versus 29.5 ± 2.96 respectively; *p* = 0.81), sex (4:6 [40%:60%] versus 0:8 [0%:100%] respectively; *p* = 0.09), pathological fractures (8 [80%] versus 4 [50%] respectively; *p* = 0.32), recurrent GCT (2 [20%] versus 0 [0%] respectively; *p* = 0.47), and mean follow-up in months (21.62 ± 5.13 versus 26.82 ± 9.48 respectively; *p* = 0.38). The parts of the proximal femur involved in denosumab and non-denosumab groups were head (0 [0%] versus 0 [0%] respectively; *p* = 1), neck (2 [20%] versus 2 [25%] respectively; *p* = 1), intertrochanteric area (4 [40%] versus 2 [25%] respectively; *p* = 0.64), and subtrochanteric area (4 [40%] versus 4 [50%] respectively; *p* = 1). All patients in the non-denosumab group had resection and arthroplasty, while 2 (20%) patients had curettage in the denosumab group only, and others had resection & arthroplasty. Intralesional curettage (20% versus 0%; *p* = 0.48) and resection and arthroplasty (80% versus 100%; *p* = 0.48) remained statistically similar between the denosumab and non-denosumab groups respectively. The results are shown in Table 1 , and the inclusion and exclusion criteria are shown in a flowchart as Fig. 3 .

**Table 1:** Baseline study characteristics comparison of denosumab with non-denosumab group

	Denosumab	Non-denosumab	*p* -value
Number of patients	10	8	–
Age in years: mean ± standard deviation	28.6 ± 5.85)	29.5 ± 2.96)	0.81
Sex: n (%)			
Male	4 (40%)	0 (0%)	0.09
Female	6 (60%)	8 (100%)	
Pathological fractures: n (%)	8 (80%)	4 (50%)	0.32
Follow-up in months: mean ± standard deviation	21.62 ± 5.13	26.82 ± 9.48	0.38
Surgical procedure: n (%)			
Intralesional curettage with hemicortical resection	2 (20%)	0 (0%)	0.48
Resection and hip arthroplasty	8 (80%)	8 (100%)	
Recurrent giant cell tumor: n (%)	2 (20%)	0 (0%)	0.47
Part of proximal femur involved: n (%)			
Head	0 (0%)	0 (0%)	1
Neck	2 (20%)	2 (25%)	1
Intertrochanteric	4 (40%)	2 (25%)	0.64
Subtrochanteric	4 (40%)	4 (50%)	1

### MSTS Score

The mean MSTS scores of the the denosumab and non-denosumab groups after 6-weeks (24 ± 6.5 versus 20.0 ± 6.0 respectively; *p* = 0.04) and 6-months (26.0 ± 5 versus 23.0 ± 0.67 respectively; *p* = 0.04) of surgery showed a statistically significant difference. On the 6-week analysis of the MSTS score, the results showed good-results in the non-denosumab group while excellent results were seen in the denosumab group. The MSTS score was excellent in both groups in the 6-month analysis and onwards. However, we have seen better pain relief and emotions ranging from the immediate postoperative phase to 6 months of follow-up after denosumab therapy, in which the mean pain score was 3.9 ± 0.4 in the denosumab group versus 2.1 ± 0.6 in the non-denosumab group. However, the mean MSTS scores were similar between the two groups at 12 months follow-up (28.8 ± 1.7 versus 29.5 ± 0.33 respectively ; *p* = 0.35). These findings are summarized in Table 2 .

**Table 2:** Postoperative comparison of outcomes between the denosumab and non-denosumab groups

	Denosumab	Non-denosumab	*p* -value
MSTS score: mean ± standard deviation			
6 weeks	24.0 ± 6.5	20.0 ± 6.0	0.04
6 months	26.0 ± 5.0	23.0 ± 0.67	0.04
12 months	28.8 ± 1.7	29.5 ± 0.33	0.35
Harris Hip score: mean ± standard deviation			
6 weeks	61.02 ± 7.36	48.52 ± 3.97	0.03
6 months	81.1 ± 2.97	79.15 ± 3.24	0.82
12 months	89.84 ± 3.75	90.05 ± 3.00	0.38
Recurrence: n (%)	0 (0%)	0 (0%)	1
Infections	0 (0%)	1 (12.5%)	0.44
Hip dislocation	0 (0%)	1 (12.5%)	0.44

**Abbreviation:** MSTS, Musculoskeletal Tumor Society.

### Harris Hip Score

The difference in the mean HHS remained statistically non-significant and excellent in the denosumab and non-denosumab groups after 6 months (81.1 ± 2.97 versus 79.15 ± 3.24 respectively ; *p* = 0.82) and 12 months (89.84 ± 3.75 versus 90.05 ± 3.00 respectively; *p* = 0.38) of follow-ups postoperatively. However, the results were considerably different between the groups at the 6-week follow-up (61.02 ± 7.36 versus 48.52 ± 3.97 respectively; *p* = 0.03), at which point good results were obtained in the denosumab group, while fair results were obtained in the non-denosumab group. Considering the pain scale of the HHS, significantly higher pain was observed in the non-denosumab group than in the denosumab group in the first 6 months postsurgery, which may be attributed to higher hip dysfunction in the non-denosumab group. However, the difference in pain became non-significant after 12 months of follow-up. These findings are summarized in Table 2 .

### Recurrence

None of the patients in any of the groups experienced recurrence ( *p* = 1), as shown in Table 2 .

## Discussion

GCT of proximal femur has not been discussed in depth in the scientific literature. According to our literature search, this study is the first study focusing upon the role of denosumab in GCT of proximal femur. Majority of the previously published articles focused upon the different types of reconstructions after resection of GCT of proximal femur. 7 We further focused upon grade III GCT of proximal femur only excluding cases of grade I and II GCT. The study was designed as a comparative study to compare the results with and without neoadjuvant denosumab. We also utilized denosumab in significantly lower dosage preoperatively as recent studies have utilized higher and longer dosing regimens for downstaging of tumor that included a loading regimen at day 0, 8, 15, then once monthly or fortnightly. 10

Surgical downstaging of GCT with the neoadjuvant denosumab and its correlation with the future choice of surgery has been discussed in the literature. Rutkowski et al. 11 concluded in their results that denosumab proved to be successful in surgical downstaging and to defer surgery in 48% participants. However, the recurrence rate was 15% while monthly doses of denosumab were used for a long time. 11 The long-term utility of denosumab has been proposed as risk factor for sarcomatous malignant changes in GCT. 12 Therefore, short-term use of neoadjuvant denosumab coupled with surgery remains the optimal and safer modality. But, the choice of surgical procedure remains the most crucial step for management.

Previously, major focus was kept on considering intralesional curettage for GCTs as a less morbid procedure, but it led to higher rates of recurrence. 13 Errani et al. 14 concluded that intralesional curettage for the proximal femur was a risk factor for recurrence. Therefore, we performed resection in 16 out of 18 patients and intralesional curettage in 2 patients. We tried curettage in 3 cases, but in 1 candidate, the calcar femorale was unsalvageable; thus, we converted to resection and arthroplasty with Wagner prosthesis. Wijsbek et al. performed a study in which they found better result with curettage than joint replacement for GCT of the proximal femur. 8 In another study by Khan et al., 6 they included customized endoprosthesis for GCT of the proximal femur. From our point of view, the site of the tumor remains a better indicator for selection between resection or curettage rather than the downstaging after denosumab.

To assess the functional and oncological outcomes, we performed analysis at 6 weeks, 6 months, and 12 months. We wanted to see gradual improvement in patients with the passage of time. The HHS has been traditionally used in sports surgery and arthroplasty. 15 According to our results, the HHS and MSTS score have remained significantly better in the denosumab group during the immediate postoperative phase. However, a rapid increase in hip functions was observed according to the HHS, thereby producing similar results after 6 months of follow-up. Significant pain improvement was reported by all candidates who used denosumab. Petranova et al. 16 proposed that significant pain relief has been reported in 53.6% of GCT candidates. Hence, pain improvement preoperatively led to better rehabilitation and functional outcome for hip joint in the denosumab group in our study.

Recently conducted studies have associated the neoadjuvant use of denosumab with increased incidence of recurrences. 17 However, it is noteworthy to mention that these studies have used denosumab with curettage where increased recurrences were reported. 13 Agarwal et al. 18 performed a study where they proposed that recurrence after denosumab and curettage might be prevented by in-depth curettage of the tumor to pretreatment margins according to pretreatment radiographs. The osteosclerotic rim provides protection to certain remnants of giant cells while curettage that leads to recurrence within first 2-years after the primary surgery. Therefore, our results have shown no incidence of recurrence in any cohort signifying that denosumab is not associated with recurrence.

Denosumab primarily forms an osteosclerotic rim around the tumor that leads to easier handling and resection of tumor that decreases the intraoperative duration as shown by Sahito et al. 19 and Muller et al. 20 The decrease in intraoperative duration lowers the chances of microbial growth, as shown by Cheng et al. 21 and Teo et al. 22 Hence, in our study, one case of deep infection developed in which revision surgery has been performed in the non-denosumab group. Moreover, the primary surgery was performed in 108 minutes, which was the longest duration among all 18 operated cases.

## Conclusion

In conclusion, denosumab has proven to be beneficial for GCT of the proximal femur by providing early rehabilitation and better surgical and oncological outcomes. However, the downstaging of GCT of the proximal femur for joint salvage of hip remains questionable, and further research is needed. Moreover, those candidates who are not willing or have certain serious adverse effects to denosumab must be counseled about slower progress and rehabilitation and early postoperative difficulties, especially pain. We also concluded that once-weekly dosing of denosumab for 4 weeks is the optimal dosage in the month preceding surgery, and higher dosage may not be necessary.
